# Experimental vaccination of sheep and cattle against tick infestation using recombinant 5′-nucleotidase

**DOI:** 10.1111/j.1365-3024.2009.01168.x

**Published:** 2010-02

**Authors:** M HOPE, X JIANG, J GOUGH, L CADOGAN, P JOSH, N JONSSON, P WILLADSEN

**Affiliations:** 1 CSIRO Livestock IndustriesSt Lucia, Queensland, Australia; 2 University of QueenslandSt Lucia, Queensland, Australia

**Keywords:** antigen, *Boophilus microplus*, *nucleotidase*, *Rhipicephalus (Boophilus) microplus*, *vaccine*

## Abstract

Limited prior evidence suggests that 5′-nucleotidase, an ectoenzyme principally located in the Malpighian tubules of the tick *Rhipicephalus (Boophilus) microplus*, could be an effective antigen in an anti-tick vaccine. To assess this, recombinant 5′-nucleotidase was expressed in *Escherichia coli* and used in vaccination trials with both sheep and cattle. Vaccinated sheep were challenged with freshly moulted adult ticks. Those with high titres of anti-nucleotidase antibodies showed significant protection against tick infestation, although protection was less than that found with the previously characterized antigen, Bm86. Cattle were vaccinated, in separate groups, with 5′-nucleotidase, Bm86 and both antigens combined. Cattle, as the natural host, were challenged with larval ticks. Although Bm86 showed typical efficacy, no significant protection was seen in cattle vaccinated with 5′-nucleotidase. Cattle receiving a dual antigen formulation were no better protected than those receiving Bm86 alone. One possible reason for the difference between host species, namely antibody titre, was examined and shown to be an unlikely explanation. This demonstrates a limitation of using a model host like sheep in vaccine studies.

## Introduction

One of the major stumbling blocks in the development of anti-tick vaccines, as with other anti-parasite vaccines, is the identification of effective antigens (1). For ticks, an existing efficacious antigen, Bm86, is the basis of two commercial vaccines, TickGARD Plus and Gavac Plus (1) directed against the cattle tick *Rhipicephalus (Boophilus) microplus*. Unless antigens yet to be discovered have efficacy as good as or better than Bm86, it is likely that additional antigens will be primarily of interest in dual antigen vaccine formulations aimed at further enhancing efficacy or duration of protection. Yet, the ability of antigen cocktails to increase vaccine efficacy has received little experimental examination and results have been equivocal (2).

The 5′-nucleotidase from *R. (Boophilus) microplus* would seem to have potential in the ‘concealed antigen’ approach to vaccination (3). The purification and characterization of the native protein have been previously reported (4,5;). Among the characteristics likely to be of importance for ‘concealed antigens’ are accessibility to antibody ingested during tick feeding and a physiological function of importance to the tick. Ideally too, one would expect limited functional redundancy, that is, the antigen should not have a large number of immunologically different variants capable of performing the same physiological function. The 5′-nucleotidases are ectoenzymes commonly used as markers for cell plasma membranes. The tick enzyme, like the protective *R. (Boophilus) microplus* antigen Bm86 (6), is bound by a glycosyl phosphatidylinositol (GPI) anchor to the membrane. In *R. (Boophilus) microplus*, the enzyme appears to be abundant on the surface of cells lining the Malpighian tubules (7) and hence potentially accessible to antibodies. It is known that antibody can persist for long periods in the tick gut and that undigested haemoglobin, and hence presumably other proteins, can pass through the tick gut and be excreted (8). Evidence from Southern blots suggests that while there is more than one nucleotidase gene in *R. (Boophilus) microplus* (9), the number is small. Its enzymatic activity is unusual, in that it degrades not only nucleotide monophosphates to nucleosides but also the di- and triphosphates (4). These activities and the location of the enzyme suggest a role in purine salvage (7) although this has not been clearly established. The importance of purine salvage to ticks is suggested by the fact that ingestion of allopurinol in an *in vitro* feeding system increased mortality (7). Allopurinol is an inhibitor of hypoxanthine guanine phosphoribosyl transferase, a component of the purine salvage pathway.

However, there has been virtually no examination of 5′-nucleotidase as an antigen. Purification of the native enzyme from semi-engorged female ticks gave amounts of protein too low for convincing vaccination trials in cattle (4). Early attempts to produce a recombinant form of the enzyme in *Escherichia coli* yielded large but incomplete fragments of the protein with variable C-terminal truncations (9). It was shown, however, that antibodies to this expressed protein as well as antibodies raised to the enzymatically active, baculovirus-expressed protein reacted with native tick protein using Western blots and immunofluorescent localization on tick organs (7). The *E. coli*-expressed fragment was trialled in combination with Bm86 in a cattle vaccination experiment. The results suggested a slight improvement in efficacy, although the animal numbers were too small and the effect too slight to show statistical significance (10).

Support for the use of 5′-nucleotidase as an antigen came from *Ixodes scapularis* (11). Using an expression library constructed using an *I. scapularis* cell line, mice were vaccinated and challenged with a tick infestation. Iteration of the process identified a number of efficacious genes, one of the best being 4F8, a fragment of 316 amino acids identified as a 5′-nucleotidase. This fragment, however, has only 18% identity with the 5′-nucleotidase from *R. (Boophilus) microplus*, suggesting that the two proteins are only distantly related.

The primary objective of current research therefore was to assess the potential of 5′-nucleotidase both alone and in combination with Bm86 as antigens to protect ruminants against tick challenge. This was performed using two experimental hosts, sheep and cattle. Sheep can be a useful host in vaccination trials (12,13;). Although larval attachment and development can be problematic, freshly moulted adult ticks attach readily, engorge and lay eggs normally (12). Whether the two hosts are equivalent, however, is an unresolved question. It was noticed, using Bm86 as an antigen, that vaccine efficacy in sheep was much higher than in cattle (12,14;). Recombinant 5′-nucleotidase was therefore assessed in both sheep and cattle to see if the results obtained with Bm86 were unique to that antigen or were also observed with a second antigen.

## Materials and methods

### Materials

#### Parasite RNA extraction and cDNA synthesis

*Rhipicephalus (Boophilus) microplus* (non-resistant field strain (NRFS)) ticks were supplied by Queensland Primary Industries and Fisheries (QPIF), Yeerongpilly. Total RNA was prepared from *R. (Boophilus) microplus* using Trizol Reagent as per the manufacturer’s recommendations (Invitrogen; Groningen, The Netherlands). For cDNA synthesis, 1 μg of total RNA was reverse transcribed in a 20 μL reaction mix using 0·5 μg oligo dT_12–18_ primer and 200 U Superscript III (Invitrogen) at 50°C for 60 min.

Oligonucleotides Nucleotid-F 5′GAATTCAACCGACTTCACGGCGACAG and Nucleotid-R 5′ GCGGCCGCGCAAGCATCCGAAGCCTGG were used to amplify the open reading frame of 5′-nucleotidase, which was cloned into the pCR2.1 TA vector (Invitrogen) and sequenced. The cDNA was subsequently sub-cloned into the pQE31 expression vector (QIAGEN, Valencia, California, USA) in frame with the N-terminal Hexa-His tag. The Bm86 gene was amplified using primers Bm86-Y 5′GCGGCCGCACTTGACTTTCCAGGATC and Bm86-Z 5′GAATTCAGAATCCATTTGCTCTGAC and cloned into the pCR2.1 TA vector and sequenced. The cDNA was subsequently cloned into pPICZαC in frame with sequence encoding the signal sequence. Sequences were aligned using the National Centre for Biotechnology Information (NCBI) pairwise Blast server.

### Isolation of antigens

The recombinant *R. (Boophilus) microplus* 5′-nucleotidase protein was produced in *E. coli* using the expression vector noted above. The recombinant protein was purified from *E. coli* inclusion bodies using Ni-NTA affinity chromatography (QIAGEN) following the manufacturer’s recommendations. Following purification, the 5′-nucleotidase protein was diluted to 1 mg/mL and added slowly to buffer (20 mm Tris, 3 m urea, 500 mm NaCl, 1 mm EDTA, 1 mm reduced glutathione, 0·2 mm oxidized glutathione, 0·5 m arginine hydrochloride, 0·01% Tween 20) to promote refolding of the protein.

The *Pichia* protein expression system was purchased from Invitrogen. Recombinant Bm86 was expressed in *Pichia* following the manufacturer’s recommendations. The *Pichia* strain, KM71H, was transformed with the pPICZαC-Bm86 plasmid using the lithium chloride method as detailed in the manufacturer’s protocol. Clones were analysed by PCR for integration of the Bm86 cDNA. Starter cultures were grown in buffered minimal glycerol containing histidine (BMGH) media until OD >2 then changed to buffered minimal methanol containing histidine (BMMH) and incubated at 28°C with shaking. Methanol was added daily for 4 days to induce and maintain recombinant protein expression. On the fourth day, the culture was centrifuged at 2000 *g* for 10 min and the supernatant collected. The supernatant was further clarified by centrifugation (6000 *g*, 4°C for 30 min) and 1 mm PMSF added. The supernatant was then further clarified by filtration through a series of 5, 2 and 0·45 μm filters.

After final clarification, the supernatant volume was reduced using a stirred cell apparatus fitted with a 50 kDa cutoff filter to a final volume of approximately 50 mL. This solution was buffer exchanged for ConA affinity purification (10 mm Tris, 0·15 m NaCl, 1 mm CaCl_2_, 1 mm MnCl_2_, pH 7·5). Affinity purification was achieved using ConA Sepharose (Pharmacia, Uppsala, Sweden). After equilibration of the column with the above buffer, the sample was loaded onto the column at a flow rate of 0·5 mL/min. The column was washed until the A_280_ returned to baseline. Bound protein was eluted from the column after a 1 h incubation with elution buffer (10 mm Tris, 0·5 m NaCl, 0·5 mα-methyl-D-mannoside, 1 mm CaCl_2_, 1 mm MnCl_2_, pH 7·5). Eluted protein was buffer exchanged to PBS.

Analysis of 5′-nucleotidase and Bm86 protein expression and the Ni-NTA and ConA affinity chromatography were performed on standard SDS-PAGE gradient gels (15) and stained with silver (16). The protein concentrations of the purified recombinant proteins were determined using the BCA protein assay (Pierce, Rockford, IL) according to the manufacturer’s instructions. Amino terminal sequencing using an Applied Biosystems 492–01 HT protein sequencer (Foster City, CA, USA) validated the amino termini of both recombinant proteins.

### Vaccination of sheep and parasite challenge

Sheep vaccinations were conducted in Border Leicester crossbred wethers. Each antigen was used to vaccinate six sheep, three using ISA50 as adjuvant and three with ISA773 (Seppic). Vaccines were prepared according to the manufacturer’s instructions, with 80 μg of the relevant antigen per injection. Two control groups received adjuvant alone.

Animals were given three intramuscular injections each 1 month apart with tick challenge occurring 2 weeks following the third vaccination. Blood samples from the jugular vein were collected at the time of each vaccination, at tick challenge and at completion of the trial.

For the tick challenge, engorged nymphs of *R. (Boophilus) microplus* were picked off previously unexposed *Bos taurus* donor cattle, each infested with 30 000 larvae of the acaricide-susceptible NRF strain, purchased from QPIF. Nymphs were held in a humidified incubator until adults emerged.

Sheep were prepared for tick challenge by closely cropping the wool along the dorsal line and gluing 2 cm internal diameter aluminium rings to shaved skin. Duplicate rings were used on each sheep. Each ring was infested with 10 female and 5 male newly emerged adult ticks, then covered with a gauze lid. Following engorgement of the adult females (∼7 days), ticks were collected and weighed. All female ticks surviving to engorgement were incubated to assess their capacity for egg laying.

### Vaccination of cattle and parasite challenge

The cattle used were *B. taurus* (Hereford × Angus) heifers, 9 months old, with no prior exposure to *R. (Boophilus) microplus.* Cattle were divided into three experimental groups, each containing four animals, and were vaccinated intramuscularly three times at monthly intervals, each with 100 μg of antigen, either Bm86 or nucleotidase. An additional group received a combination of the two proteins, using 100 μg of each. Each antigen preparation was formulated in a two component adjuvant. Half of the antigen was formulated in ISA50 in the usual manner, while the other half was formulated in 10 mg/mL QuilA. The final vaccine was prepared by mixing nine volumes of ISA50 formulation with one volume of QuilA. Four control cattle received adjuvant alone. Blood was sampled from the jugular vein at six time points: at the time of each vaccination, before larval challenge, on day 19 of the infestation, that is just before the engorgement of the first adult ticks and on termination of the experiment.

Cattle were infested daily with 1000 tick larvae for 21 days, beginning 1 week following the third vaccination. After the last infestation, cattle were moved into individual moated pens and for the following 3 weeks, all engorged adult female ticks dropping daily from the host were collected, counted, weighed and visually examined for damage. The results listed in Table 1 are the means of the data for 18 individual days. On days 2 and 3, 6–10 and 14 of this collection period, a sample of 50 females were weighed and incubated to assess egg laying capacity. Reproductive potential was calculated as the product of the mean number of ticks engorging per day, their weight and their fecundity. Effectively it measures the mass of eggs laid by the female ticks engorging from an infestation of 1000 larvae.

**Table 1:** Vaccination of cattle with recombinant 5′-nucleotidase and Bm86 separately and as a dual antigen vaccine

Antigen	Animal	Mean tick no.	Mean wt. (mg)	Fecundity	Reproductive potential
5′-Nucleotidase	1	316 ± 94	265 ± 25	0·54 ± 0·04	45·2
	2	210 ± 86	258 ± 17	0·52 ± 0·04	28·2
	3	272 ± 104	233 ± 30	0·36 ± 0·18	22·8
	4	358 ± 137	260 ± 29	0·51 ± 0·04	47·5
	Mean	289 ± 63	254 ± 14	0·48 ± 0·08	35·9 ± 12·3
Bm86	5	142 ± 45	183 ± 16	0·13 ± 0·05	3·4
	6	160 ± 56	186 ± 9	0·32 ± 0·06	9·5
	7	175 ± 53	194 ± 15	0·30 ± 0·08	10·2
	8	88 ± 26	156 ± 27	0·17 ± 0·05	2·3
	Mean	141 ± 38**	180 ± 17**	0·23 ± 0·09**	6·4 ± 4·1**
Dual antigen	9	224 ± 67	192 ± 12	0·32 ± 0·07	13·8
	10	154 ± 42	182 ± 12	0·21 ± 0·11	5·9
	11	198 ± 69	193 ± 7	0·29 ± 0·13	11·1
	12	221 ± 74	180 ± 12	0·31 ± 0·15	12·3
	Mean	199 ± 32*	187 ± 7**	0·28 ± 0·05**	10·8 ± 3·4**
Control	13	256 ± 119	234 ± 24	0·52 ± 0·03	31·2
	14	357 ± 107	246 ± 23	0·52 ± 0·05	45·7
	15	234 ± 102	267 ± 27	0·49 ± 0·05	30·6
	16	277 ± 56	251 ± 15	0·35 ± 0·05	24·3
	Mean	281 ± 54	250 ± 14	0·47 ± 0·08	33·0 ± 9·1

Tick numbers and weights are the means of 18 measurements; reproductive potential the mean of 8.

Means significantly less than controls: *P< 0·05; **P< 0·01.

Statistical analyses were performed using Sigma Stat.

### Measurement of antibody titre

Antibody titres were measured in serum against the relevant antigen using ELISA (17,18;). Briefly, antigen was coated on wells of ELISA plates at a concentration of 1 μg/mL, then overlayed with serial 2-fold dilutions of sheep or bovine sera starting at an initial dilution of 1 in 1000. Peroxidase-coupled rabbit anti-ovine IgG and ovine anti-bovine IgG were used as second antibodies at a dilution of 1 in 1000. A pool of sera from a sample taken prior to tick challenge and at maximum antibody titres was used as an internal standard in all ELISA assays, while pre-vaccination sera acted as negative controls on all plates.

A series of assays to compare ovine and bovine anti-nucleotidase titres were performed in an identical way, with the substitution of peroxidase-coupled protein G (Thermo Scientific, Waltham, MA, USA) as the detection reagent. Dilutions used were 1 in 500, 1000 and 2000 of a 1 mg/mL solution prepared according to the manufacturer’s instructions.

The ability of ovine IgG to compete with bovine IgG in binding to nucleotidase on an ELISA plate was examined by preparing serial 2-fold dilutions from 1 in 2000 to 1 in 512 000 of the standard bovine anti-nucleotidase pool in the presence of a 1 in 2000 dilution of the ovine reference pool. The same bovine dilution series was also prepared in a 1 in 4000 dilution of the ovine serum, and in increasing dilutions of the ovine serum to a maximum of 1 in 64 000. ELISA assays were conducted as usual, with peroxidase-coupled ovine anti-bovine IgG as a detection reagent. To minimize and chance that the ovine anti-bovine IgG detection reagent would react with ovine IgG bound to the ELISA plate, the second antibody was diluted in 1 in 1000 normal ovine serum.

## Results

### Preparation of 5′-nucleotidase and Bm86

The 5′-nucleotidase and Bm86 cDNAs were derived from the NRFS tick isolate, whereas the original proteins and cDNA sequences were from the Yeerongpilly or Y isolate. The 5′-nucleotidase sequence, however, showed only two amino acid substitutions, one conservative. The Bm86 sequence contained 14 amino acid changes distributed through the coding sequence, a variation of 2·5%. This is typical of the allelic variation seen within this protein in Australia (14). The recombinant proteins were purified as detailed in Materials and methods section and analysed by SDS-PAGE. The sizes of the recombinant proteins were approximately 65 and 100 kDa for 5′-nucleotidase and Bm86 respectively. N-terminal amino acid sequencing of the purified proteins confirmed their identity.

### Vaccination of sheep

Groups of six sheep were vaccinated with each of the recombinant antigens, 5′-nucleotidase and Bm86, or with adjuvant alone. Within each group of six, three were vaccinated using ISA50 as adjuvant and three using ISA773. Geometric mean antibody titres for the two antigens in two adjuvants in 12 sheep are listed in Table 2. Two way analysis of variance of log_10_ transformed titres using antigen and adjuvant as factors showed the effect of adjuvant to be significant (*P*< 0·01), average peak antibody titres being 5-fold lower with ISA773. An experiment with recombinant Bm91 antigen in the two adjuvants showed the same disparity in mean antibody titres (P. Willadsen, unpublished data).

**Table 2:** Effects of adjuvant on geometric mean antibody titres in sheep

	Adjuvant		
Antigen	ISA50	ISA773	Difference (fold)
5′-Nucleotidase	75 000	14 700	5·1
Bm86	62 400	12 100	5·2

Efficacies of the 5′-nucleotidase and Bm86 vaccines were assessed with an adult tick challenge as described in Materials and methods using female ticks in a ring attached to the shaved skin of sheep, and duplicate rings on each sheep. Engorgement success was calculated relative to the number of female ticks attached 24 h after application, rather than the number applied within each ring initially. The number of survivors at 24 h was used as the baseline because damage to ticks is always possible in the process of physical removal of engorged nymphs from their bovine host, moulting in an incubator and re-application to sheep. However, survival and attachment at 24 h were high, an average of 89% of the ticks applied and there was no significant difference between ticks on control (88%) and vaccinated sheep (92% for 5′-nucleotidase; 87% for Bm86). Therefore, accidental damage was not important and neither was early vaccine-induced mortality significant.

Three parameters were used to measure feeding success: percentage of ticks completing engorgement relative to ticks attached at 24 h; fecundity measured as mass of eggs laid relative to the mass of engorged ticks and, as a single, summary indicator of vaccine efficacy, the reproductive potential measured as the mass of eggs laid per 10 ticks attached at 24 h. The results are listed in Table 3. Vaccination results were analysed using a two way anova with treatment (vaccinated or control) and adjuvant (ISA50 or ISA773) as factors, adjuvant acting as a *de facto* measure of antibody titre, given the large differences between mean antibody titres obtained with the two adjuvants. The significance of pairwise differences between groups was examined using the Tukey Test.

**Table 3:** Vaccination of sheep with recombinant 5′-nucleotidase and Bm86

		Parasitological parameters		
Antigen	Adjuvant	Engorgement (%)	Fecundity	Reproductive potential
5′-Nucleotidase	ISA50	29·3 (*P*< 0·01)	34·2	143 (*P*< 0·05)
Bm86		18·3 (*P*< 0·01)	27·0 (*P*< 0·05)	77 (*P*< 0·01)
Control		70·8	66·9	526
5′-Nucleotidase	ISA773	71·1	84·9	637
Bm86		14·5 (*P*< 0·01)	12·5 (*P*< 0·01)	44 (*P*< 0·01)
Control		73·2	88·6	588
5′-Nucleotidase	Combined	50·3	59·6	390
Bm86		16·4 (*P*< 0·01)	19·8 (*P*< 0·01)	61 (*P*< 0·01)
Control		73·2	77·7	557

Where no probability is given, the difference between the vaccinated and control groups was not significant at *P*< 0·05.

Fecundity = (mass eggs laid/mass engorged ticks) × 100.

Reproductive potential = mass eggs laid (mg)/10 female ticks attached at 24 h.

**Figure 1:**
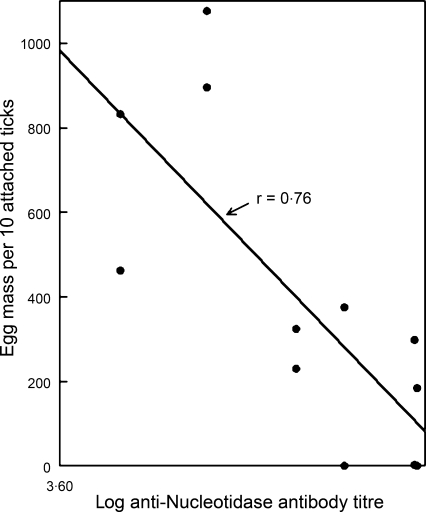
Correlation of the antibody titre to 5′-nucleotidase with the mass of eggs laid per 10 female ticks attached to sheep after 24 h.

The effect of vaccination with Bm86 was significant for all parameters (*P*< 0·01) with both adjuvants separately and as a combined group. For the group vaccinated with 5′-nucleotidase, the effect of adjuvant was significant for percentage engorgement (*P*< 0·01), fecundity (*P*< 0·05) and reproductive potential (*P*< 0·01). Despite the small animal numbers, the effects of 5′-nucleotidase were significant when ISA50 was used as adjuvant. In neither the group vaccinated using ISA773 nor in the combined group was the effect on ticks significant. The likely explanation is the differences in antibody titre. The probable importance of circulating antibody level was reinforced by examination of the correlations between the parasite parameters and log_10_ antibody titre for all sheep (Figure 1). All correlations were statistically significant (*P*< 0·01).

### Vaccination of cattle with recombinant 5′-nucleotidase alone and in combination with Bm86

Groups of four cattle were vaccinated with recombinant 5′-nucleotidase, Bm86 and the two antigens in combination and challenged with a standardized infestation of larval ticks as described in Materials and methods. The results of parasite challenge are listed in Table 1. The Bm86 antigen gave overall protection, measured by the reproductive potential, of 81% with statistically significant effects on each of the individual parameters of mean numbers of ticks engorging, mean weight of engorged female ticks and mean fecundity (all *P*< 0·01). In contrast, vaccination with 5′-nucleotidase had no effect on any parameter. As it is always possible that two antigens in combination are more efficacious than either singly, the effect of combining the antigens was also investigated. The data in Table 1 show, however, that the dual antigen combination had no advantage compared with Bm86 alone. In fact, overall protection in the combined group was decreased by about half compared with Bm86 alone, although the difference was not statistically significant.

### Comparative antibody responses of sheep and cattle

**Figure 2:**
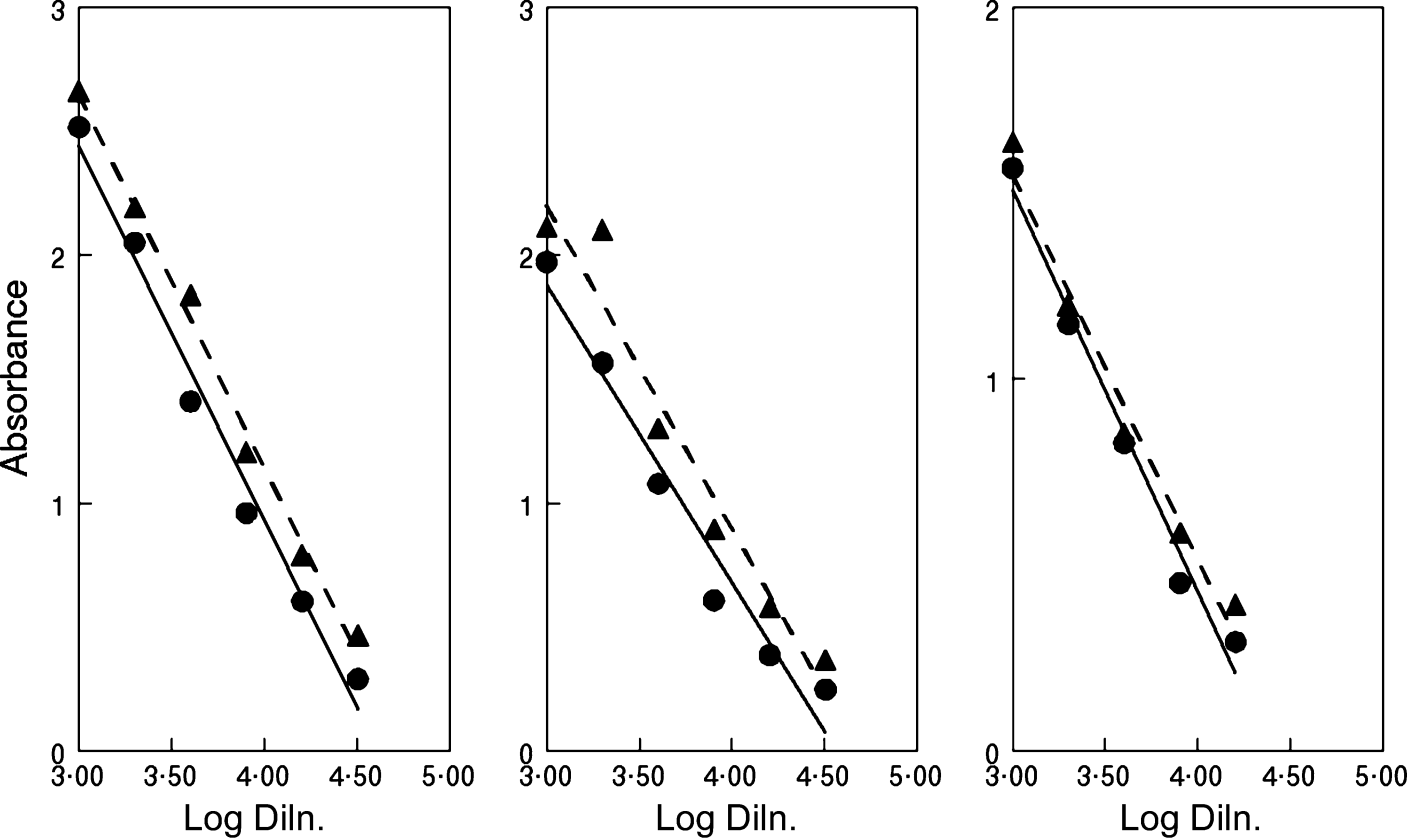
ELISA assays of pooled, high titre bovine (•) and ovine (▴) sera against recombinant 5′-nucleotidase. The detection reagent was peroxidase-coupled protein G at dilutions of (from left to right) 1 in 500; 1 in 1000 and 1 in 1500.

The fact that 5′-nucleotidase, at least at high antibody titres, seemed to be quite efficacious against tick infestation in sheep but completely ineffective in cattle is important to understand. For sheep, the evidence suggests that efficacy may correlate with antibody titre and this mirrors the experience of large numbers of trials with the Bm86 antigen in cattle. Therefore, one obvious explanation of the difference in protection between sheep and cattle would be a difference in anti-nucleotidase antibody titres. This was examined in two ways. In both experiments, the antisera used were pools of ovine and bovine sera of peak antibody titres. In the first experimental approach, ELISAs were performed as described in Materials and methods using recombinant 5′-nucleotidase as antigen and peroxidase-coupled protein G as the reagent to detect bound IgG. Protein G reacts strongly with both ovine and bovine IgG and so may minimize differences in the sensitivity of species-specific second antibody reagents. Peroxidase-protein G was used at three dilutions. The results are shown in Figure 2 and suggest that there was little difference in the titres of ovine and bovine anti-nucleotidase antibodies.

**Figure 3:**
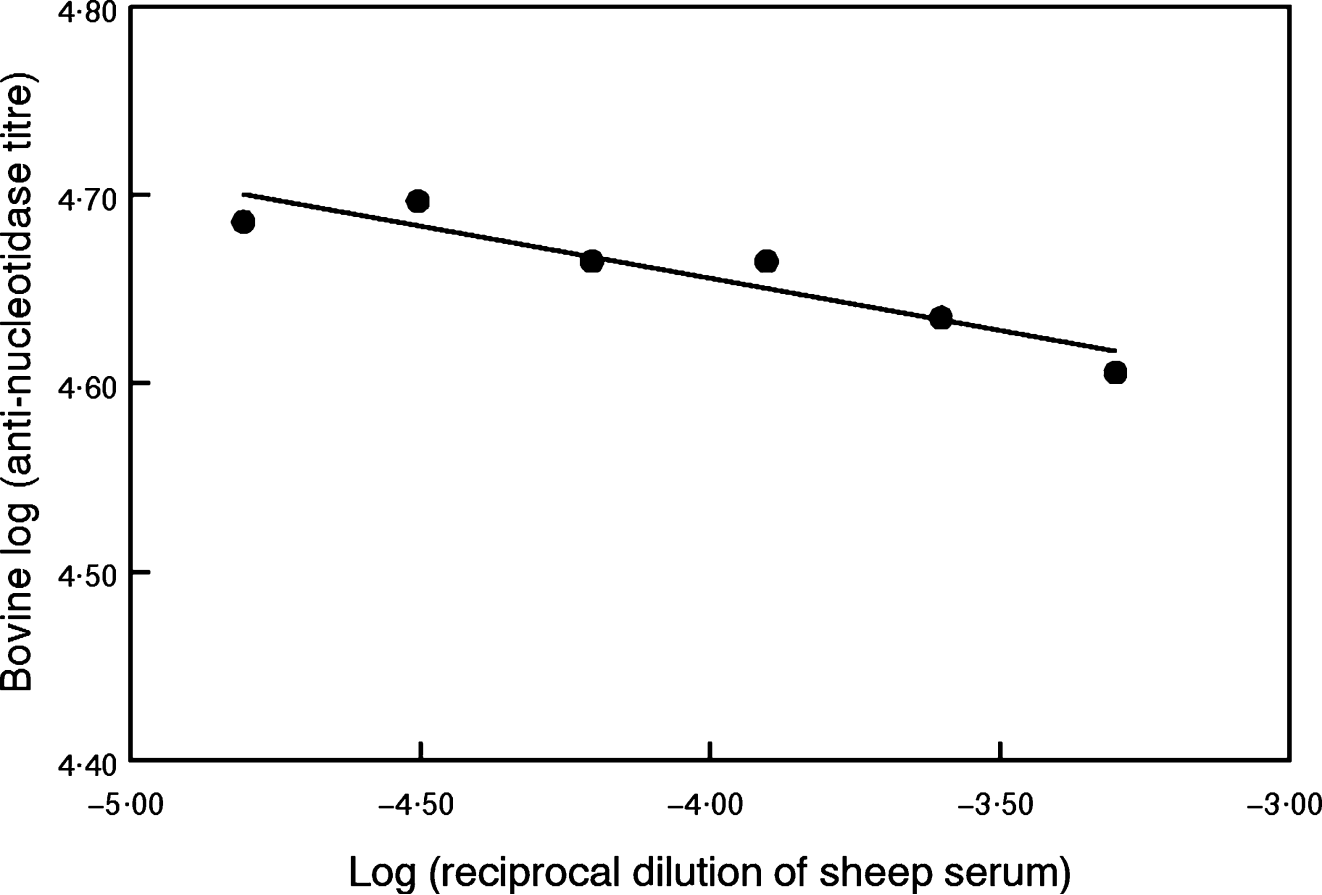
Log titres bovine antibody to 5′-nucleotidase, measured as described in Materials and methods, in the presence of varying dilutions of ovine anti-5′-nucleotidase. The detection reagent was ovine anti-bovine IgG antibody coupled to peroxidase. The log titre in the absence of any ovine antiserum was 4·7.

In a second experimental approach, serial dilutions of bovine anti-nucleotidase serum were made in the presence of dilutions of ovine anti-nucleotidase ranging from 1/2000 to 1/64 000. Normal ELISAs were then performed with these dilutions, using as a second antibody peroxidase-coupled ovine anti-bovine IgG. This therefore measured the ability of an excess of ovine anti-nucleotidase to compete with bovine antibody for bound nucleotidase. As expected, there was no reaction of the second antibody with ovine IgG. The results are shown in Figure 3 and demonstrate that the competition of the ovine anti-nucleotidase with the bovine equivalent was even less than expected. For example, a 1/2000 dilution of ovine antibody reduced the binding of a 1/50 000 dilution of the bovine antibody by only 12%.

## Discussion

Successful vaccination against the tick *R. (Boophilus) microplus* has been previously achieved using the Bm86 antigen that relies on the reaction between ingested host antibody and the antigen, which is primarily located on the microvilli of the gut digest cells (14). The discovery of that antigen was the result of a laborious, expensive and time consuming process of protein fractionation, followed by vaccination trials in cattle. Improvements in the existing vaccine are likely to rely on the discovery of other antigens that, in combination with Bm86, offer enhanced efficacy. The discovery of efficacious antigens, however, remains a significant challenge. It is attractive to think that the process could be expedited in two ways. First, compared with the laborious process of using animal vaccination trials to sort through gene libraries or complex protein extracts, the use of various forms of circumstantial evidence to suggest proteins that might be effective antigens would be rapid and cost effective, if successful. Secondly, the use of a model host for vaccination trials would circumvent some of the expenses and constraints of cattle vaccination. The experiments reported here attempt to address both issues.

As summarized in the Introduction, previous research has given a number of reasons for thinking that the 5′-nucleotidase could be a protective antigen. Initial trials with recombinant 5′-nucleotidase were carried out using sheep as a model host. Sheep are a good host for adult ticks and the gross biology appears to be normal i.e. engorgement success is high and adult weights and egg laying are normal. These vaccination trials used two adjuvants, ISA50 and ISA773. The purpose of this was 2-fold. Efficacy of Bm86 vaccination in cattle is strongly dependent on antibody titre (19–21) as it probably is in sheep (12) and it was of interest to induce a range of antibody titres to 5′-nucleotidase to see if a similar relationship occurred. Secondly, ISA773 is a newer adjuvant with the potential advantage of very simple formulation under difficult field conditions, the ultimate objective of this research.

Vaccination with the recombinant Bm86 and 5′-nucleotidase antigens showed a strong effect of Bm86 on engorgement success and oviposition, with an overall efficacy of 85%. This is consistent with earlier reports, although the effect of vaccination was considerably weaker in the current experiments (12). 5′-Nucleotidase also showed good efficacy, with an overall reduction in the mass of eggs laid by a standard number of infesting adult ticks of 73%. This was seen, however, only in a group with high antibody titres. There appeared to be a significant correlation between efficacy and antibody titre, as has been reported for Bm86 in cattle (19).

Given the positive result of vaccination in sheep, it was disappointing that in a subsequent trial in cattle, the recombinant 5′-nucleotidase antigen showed no effect. In principle, it is still possible that an antigen, although ineffective on its own, may be capable of enhancing the efficacy of a combined vaccine. This is particularly the case for an antigen capable of complementing Bm86. One of the major effects of vaccination with Bm86 is to produce destruction of tick gut cells and hence greatly increased leakage of bovine antibody into the tick’s haemolymph (14). It is possible therefore that access of antibody to a second target, such as 5′-nucleotidase, would be facilitated in a dual antigen vaccine. However, there was no evidence at all for increased efficacy of a Bm86 plus 5′ nucleotidase vaccine compared to Bm86 alone. It is important although that the number of examples of experimental anti-parasite vaccination where multi-antigen formulations have actually been shown to give increased protection is very small indeed (2).

One obvious explanation of the difference between results with sheep and cattle would be a difference in anti-5′-nucleotidase antibody titre. This was examined in two ways: by a simple comparison of ELISA titres using labelled protein G as the detection reagent, on the assumption that protein G would react with both ovine and bovine IgG and secondly, in a competitive ELISA. Neither experiment supported the idea that a difference in bovine cf. ovine antibody was responsible for the vaccination result. While neither experiment may realistically reflect the binding of host IgG to nucleotidase in tick tissues, other explanations for the difference between tick host species must be sought. In practical terms, the result, combined with that reported earlier for Bm86 vaccination, suggest that sheep will be an imperfect model host for the development of an anti-tick vaccine in cattle.
